# Early health technology assessment of a primary care tool for the diagnosis and management of headaches in Alberta, Canada

**DOI:** 10.1017/S0266462326103547

**Published:** 2026-02-20

**Authors:** Sarah Koles, Diepreye Ayabina, Sasha van Katwyk, Brian Chan, Charles Yan

**Affiliations:** 1 Cumming School of Medicine, University of Calgary, Canada; 2 Institute of Health Economics, Canada

**Keywords:** early health technology assessment, headaches, eConsult, lasoo health, wait times, outcomes, productivity

## Abstract

**Background:**

Lasoo Health is an e-software in the early stages of development, designed to generate a consult report with the most probable headache diagnosis and treatment plan from direct patient input. A patient accesses the program with their consent and a referral from primary care. Digital consult is then reviewed by a medical specialist and then sent to the primary care provider to initiate care. This early health technology assessment (eHTA) assesses the potential impact of Lasoo Health on timely access to effective headache management, cost savings, and health outcomes compared to the current standard of care (SOC) in Alberta, Canada.

**Methods:**

We developed a discrete event simulation (DES) of headache diagnostic pathways for Albertan patients suffering from headaches. The model was parameterized using secondary data sources identified via relevant published literature and subject matter expert opinion. Cost-effectiveness was expressed from a societal perspective using the incremental net monetary benefit (iNMB) of Lasoo Health incorporated with the SOC compared to SOC alone over an analytical time frame of five years.

**Results:**

Our analysis suggests that incorporating Lasoo Health into the SOC may reduce specialist assessment wait times by 70 percent and total per-patient costs by 7 percent. Using a willingness-to-pay (WTP) threshold of Canadian dollars (CAD) 50,000 (U.S. dollars (USD) $35,240), the iNMB per patient was estimated to be CAD 1,069 (USD $753).

**Conclusions:**

The benefits of implementing Lasoo Health over a 5-year period could translate to improved patient outcomes, reduced wait times for specialists, and lower productivity losses among headache patients.

## Introduction

Headaches are one of the most common presentations to primary care with a lifetime prevalence of 66 percent (1). Migraines affect 2.6 million women and 1 million men in Canada. 90 percent of migraine sufferers report moderate to significant pain, with 75 percent reporting impaired function and 33 percent reporting bed rest during an attack. It is estimated that headaches account for 20 percent of work absences, a significant economic impact (2). The International Classification of Headache Disorders (ICHD) classifies headaches into primary and secondary types. Primary headaches have no known underlying cause and include migraines, tension-type headaches, and cluster headaches. Secondary headaches are a result of other conditions such as medication overuse, infection, or trauma. More than 90 percent of headache patients who present to their primary care provider have a primary headache disorder. (3).

Primary care providers who feel competent to diagnose headache patients may recommend a treatment option and/or refer headache patients to be seen by a specialist to provide a more comprehensive assessment and treatment. Current specialist referrals are fragmented and time-consuming for primary care physicians. In Canada, patients suffering from headache disorders may wait for up to 4 years to be seen by a specialist (4;5). In Alberta, wait times to be seen by a specialist are 12–18 months depending on the referral pathway (6;7). Such prolonged specialist wait times are associated with adverse consequences such as increased pain, poor medical outcomes, and increased costs from lost wages and productivity (8;9).

Lasoo Health, a digital health company, created a medical algorithm-driven software to improve access to specialists for the diagnosis and management of headaches. Their program is designed to offload the primary care physician and instead engage the patient. Once a primary care provider completes a simple electronic referral and confirms patient consent to being contacted electronically, the patient completes a Web-based, software-enabled intake questionnaire, which derives the most probable diagnosis and treatment plan for the patient. The generated consult report is reviewed by a specialist and then sent to the primary care provider to administer the care plan. By conducting a patient assessment via Lasoo Health intake forms, some patients avoid an in-person specialist visit allowing specialists more time to attend to other patients. This is projected to reduce the overall wait times for specialist assessments.

In the past few decades, there has been an increase in the use of iterative health economic modeling in the early stages of health technology development and implementation (10). These early health technology assessments (eHTAs) help provide evidence-informed value assessments and actionable insights for stakeholders on the technology’s readiness and next steps in evidence generation and product development (11). Although the current evidence on Lasoo Health remains limited, eHTA provides an opportunity to estimate its cost-effectiveness using the available data and extrapolating outcomes. As more data become available over time, these early models can be refined and validated, ensuring their relevance and accuracy in informing future healthcare policies. We present an eHTA of Lasoo Health as an intervention tool for headaches, estimating its impact on the specialist wait times and determining its economic impact.

## Methods

A model-based decision analytic approach was used to estimate the costs and benefits of including Lasoo Health in the standard-of-care (SOC) pathways for headache patients. A Canadian societal perspective was adopted in this analysis to capture the substantial cost burdens of the health system and those privately incurred by the patient. All costs are reported in Canadian dollars (CAD), but a conversion to U.S. dollars (USD) is also provided, using a conversion rate of CAD 1 = USD 0.7048 (12). The primary outcomes are the mean wait time for specialist assessment (defined as the mean duration between patients’ referral and their subsequent assessment by a specialist), the per-patient total costs, total primary provider’s visits, total emergency department (ED) visits, the total quality-adjusted life-years (QALYs), and net monetary benefit (NMB). A willingness-to-pay (WTP) threshold of CAD 50,000 (USD 35,240) was adopted, and all costs and QALYs were discounted at a 1.5 percent rate following the most recent guidelines for economic evaluation by Canada’s Drug Agency (CDA) (13).

### Intervention and comparator

The SOC (comparator) is the usual pathway a headache patient would take to get effective treatment, which may be when they visit their primary care provider or when referred to a specialist by the primary care provider. The intervention of interest, referred to as “Lasoo + SOC,” involves offering Lasoo Health at the primary care level. Patients may decline the use of Lasoo Health. If a patient consents, the primary care provider completes a brief electronic referral, and the patient would be contacted electronically to complete a Web-based software-enabled questionnaire, which takes approximately 10 minutes. Based on the input provided, Lasoo Health derives the most probable diagnosis and treatment plan and generates a summarized consult report for the specialist for review. The specialist approves or edits the consult and then sends to the primary care provider to administer. Very rarely, the specialist may request further information or an in-person visit.

### Model structure

**Figure 1.**
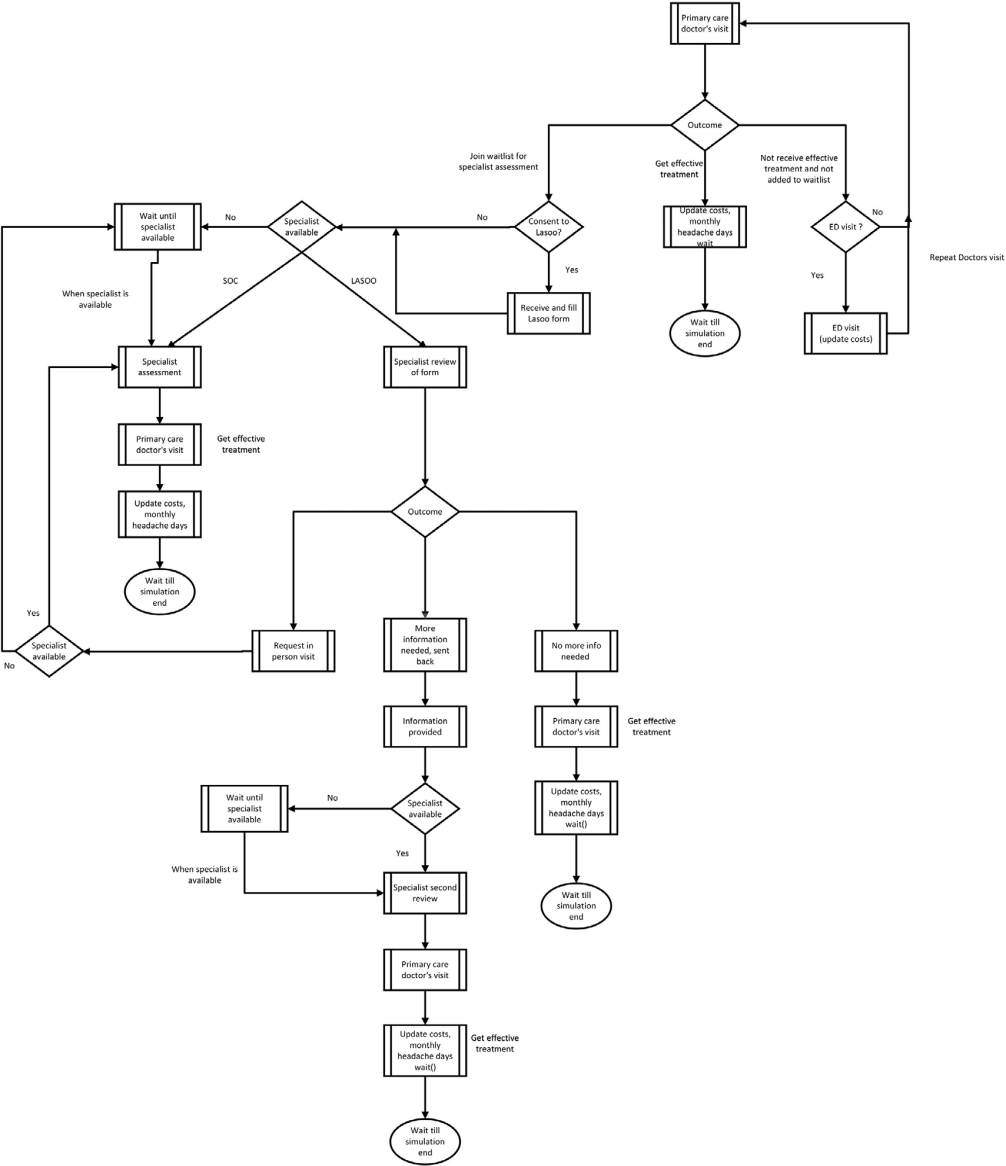
Model pathways for patients with headaches with Lasoo included at the primary care level.

A discrete event simulation (DES) model (Figure 1) was developed to examine the impact of including Lasoo Health technology in the headache care pathways in Alberta. A DES approach was selected to appropriately capture the interrelated impact of referral rates, constrained specialist resources, and changing wait time effects on patient outcomes (14). This modeling has been used to study patient flow, appointment scheduling, and operational flow in health care (15;16) as it provides a framework to evaluate outcomes such as wait times and resource utilization (15;17;18). Our model captures wait times for specialist referral, patient-level heterogeneities such as age, and type of headache experienced. We did not focus on specific treatment plans or approaches for patients with headaches, as this could be highly variable. Instead, we broadly divided treatment plans into two categories where patients are effectively managed (EM) or not EM (NEM). We assume that every headache patient enters the model as NEM, and when a patient receives an effective treatment plan, the number of days they experience headaches in a month also known as monthly headache days (MHDs) is reduced, which, in turn, leads to a reduction in the number of primary care provider’s/ED visits and missed workdays.

Starting from a visit to their primary care provider, patients could either receive an effective treatment plan or be referred for a specialist assessment. If not appropriately managed, they may continue to have future healthcare visits. For patients who are referred to see a specialist, patients may have immediate access to the specialists, or they are put on a waitlist, during which time the health system and patient may incur costs.

### Model parameters

The model was parameterized using secondary data sources identified through a search of relevant literature combined with information obtained from discussions with subject matter experts. This included consultations with practicing neurologists in Alberta where they were presented with the model structure, model assumptions, and a list of questions about headache management in Alberta. The subject matter experts reviewed model assumptions and provided estimates for some model inputs shown in Table S1. Parameters from literature were chosen such that Canadian evidence was prioritized where available and based on their applicability to the condition and the health system of Alberta, Canada. Model parameters are presented in Table S1.

### Resource capacity and availability

Based on expert opinion, there are about 100 neurologists in Alberta; however, only about 50 percent of them assess patients with headaches. Work hours are weekdays between 8 am and 4 pm during which a specialist sees on average 15 patients per day based on 30–45-minute appointments. General neurologists and headache specialists also attend to nonpatients with headaches, so it was assumed that 50 percent of visits are headache assessments. The number of primary care providers in Alberta was informed from a descriptive demographic study from 2018 to 2022 (19).

### Distribution of headache types and monthly headache days

The model assumes the most common types of headaches: migraines, tension-type headaches, cluster headaches, and secondary headaches, with distribution based on a study on patients with headaches in the United States (20). MHDs for each type of headache were informed from a variety of sources (21–23).

### Referral and consent to Lasoo Health

In this analysis, it was assumed that headache referrals are initiated at the primary care level only. For primary headache, a referral rate of 2 percent from the primary care level was assumed, based on a study on referral rates for patients with headaches (24). Canadian guidelines for primary care management of headaches (2) recommended that patients presenting with known secondary headaches be referred to a specialist. For this small subset, a 100 percent referral rate was assumed.

Patients who are referred to a specialist may be offered evaluation via Lasoo Health if they provide their consent. Based on data from a similar eConsult software used in Ontario (25;26), in the base case of the analysis, it was assumed that 75 percent of referred patients would consent to be assessed by Lasoo Health. Among patients who are not referred, a proportion of them may receive an effective treatment plan at the primary care level. However, a larger proportion are NEM and may continue to have future primary care provider or ED visits.

### Utilities

The utility values for patients with headaches are assumed to depend on their MHD. This was informed by the analysis by Mistry et al. (27), who converted the responses to the EQ-5D-5L questionnaire from a randomized trial of an intervention for people with chronic headaches into health state utilities.

### Costs

Implementation costs for Lasoo Health were not included, and it was assumed that all other associated costs are covered by existing fees within the Alberta public health system. The costs in the model include the cost of resource use (primary care provider’s visit, specialist visit, specialist review of the Lasoo Health intake, ED visit), the annual cost of effective treatment strategies, and productivity losses. The treatment cost for each patient was calculated for the total time from when they received an effective treatment plan to the end of the simulation. The cost of each primary care provider, specialist, or ED visit was based on the current rates for these services found in the Alberta Medical Association fee navigator (28). The cost of a specialist review of the Lasoo Health intake, probable diagnosis, and treatment plan was based on the current rate for eConsults in Alberta (24). The cost of treatment approaches was informed by the CDA reimbursement recommendation tables for prophylactic and acute treatment medications for migraine (29;30). Productivity losses were based on the number of missed days from work, which was informed from a Canadian study on the economic burden of migraine (22). For each patient, the cost of lost productivity/days as a result of headaches was calculated by multiplying the total number of missed days of work by the average daily wage for an adult in Canada divided by the average number of workdays per year (31;32).

### Effectiveness of treatment approaches to different headache types

We assume that effective treatment approaches reduce MHD and the number of missed workdays by the same percentage. The effectiveness of headache treatments was based on published literature and shown in Table S1.

### Arrivals, service times, and time lag

The frequency of patients with headaches arriving at primary care for a provider’s visit was described using interarrival times of an exponential distribution, defined as the time (in days) between successive arrivals. The arrival rate of this distribution was informed from data on the number of patients Albertan primary care providers see in a day (33), adjusted by the proportion of these patients that are expected to be patients with headaches (34). Service times were assumed to follow a gamma distribution. More details of the parameters used in the model are provided in Table S1.

### Scenario analyses

A series of scenario analyses was run, varying key model parameters to evaluate the impact on the results. These scenarios were developed based on consensus among the research team members on the parameters that we believe are the most relevant sources of uncertainty and the biggest structural drivers that may result in variability in model outcomes. Keeping all other model parameters constant, the following scenarios were considered:

1. Scenario 1: Referred patients who consent to Lasoo Health was set to 50 percent.
2. Scenario 2: Referred patients who consent to Lasoo Health was set to 95 percent.
3. Scenario 3: Lasoo Health reviews that require more information were set to 50 percent.
4. Scenario 4: Lasoo Health reviews that require an in-person specialist visit were set to 50 percent.
5. Scenario 5: The proportion of a specialist’s workday that is available to patients with headaches’ appointments was set to 25 percent ensuring the mean wait time for SOC matched the observed wait times in Alberta.
6. Scenario 6: The referral rate for primary headaches was set to 30 percent.
7. Scenario 7: The effectiveness of headache treatments was set to 25 percent.

### Simulation

The DES model was implemented using the Simmer package (35) in the R language and environment for statistical computing (36). The time unit was in days, and the simulation horizon was 1,826.25 days (5 years). This duration was chosen as parameters such as the number of specialists were fixed throughout the simulation, and longer time frames would have compromised the accuracy of this assumption. To replicate the wait times observed in Alberta SOC, the arrival rate of patients with headaches in the model was used as a calibration variable, leaving all other model parameters constant. New patients continuously enter the simulation for the duration of the model run time, and as such, the total time spent in the simulation is different for each headache patient. A single realization of the model was run for each of the SOC and Lasoo + SOC, and then, the mean and standard deviation of model outcomes per patient in the simulation for both strategies and the difference between them were reported. A total of 50 independent replications of the model were run for each strategy, and the mean and standard deviation of model outcomes were reported across all replications (Table S3) to understand the impact of sampling on model results.

## Results

The base case model results are included in Table 1. The mean wait time per patient in SOC was estimated to be 556 days. The associated mean total costs per patient were estimated to be CAD 37,881 (USD 26,698). More than ninety percent of this cost was attributable to productivity losses because of missed workdays per patient, which was estimated to be 149 days. Lasoo + SOC, on the other hand, was associated with a shorter (71 percent less) average wait time of 162 days, a total cost per patient of CAD 36,849 (USD 25,971), and missed workdays per patient of 135 days. Compared to SOC, Lasoo + SOC is estimated to lead to 0.22 less ED visits per patient and 0.04 fewer primary care provider’s visits per patient, save CAD 26.61 (USD 18.75) in visit costs per patient, and gain a very small (0.0007) improvement in QALYs per patient. Lasoo + SOC is also associated with an MHD of 8.50, and at the end of the simulation, 71 percent of patients with headaches are estimated to be on effective treatment plans. SOC was associated with a higher mean MHD of 9.46, and a smaller proportion of patients with headaches are estimated to have received effective treatment at the end of five years. Results from a healthcare perspective are also presented in Table S2.

**Table 1.** Model outcomes for Lasoo Health, SOC, and the difference between them for the base case analysis

Outcome	Lasoo + SOC		SOC		Difference	
	Mean	SD	Mean	SD	Mean	SD
Average wait time (days)	162.68	110.13	555.54	313.81	−392.87	332.57
Mean headache days per month (days)	8.50	4.38	9.46	4.76	−0.96	6.47
Percentage of cohort effectively managed (%)	70.91	-	53.23	-	17.68	
Mean number of primary care provider visits per Patient	7.44	5.69	7.48	5.49	−0.04	7.91
Mean number of ED visits per patient	6.19	5.51	6.41	5.34	−0.22	7.67
Mean total missed workdays per patient	134.94	134.03	149.18	146.64	−14.24	198.67
Mean treatment costs per patient ($)	8,047.85	10,259.50	6,123.44	9,756.10	1,924.41	14,157.64
	(5,672.12)	(7,230.89)	(4,315.80)	(6,876.09)	(1,356.32)	(9,978.30)
Mean productivity losses per patient ($)	27,924.80	26,965.11	30,853.73	29,585.23	−2,928.93	40,030.02
	(19,681.39)	(19,005)	(21,745.71)	(20,851.67)	(−1,454.93)	(28,213.16)
Mean total costs per patient ($)	36,849.42	30,666.35	37,880.55	32,539.57	−1,031.13	44,712.96
	(25,971.47)	(21,613.64)	(26,698.21)	(22,933.89)	(−726.74)	(31,513.69)
Total costs ($)	119.4 billion	-	122.5 billion	-	−3.2 billion	-
	(84.1 billion)		(86.4 billion)		(−2.3 billion)	
Mean total QALYs per patient	1.44	0.78	1.44	0.80	0.00	1.12
Total accrued QALYs	4.7 billion	-	4.7 billion	-	7,976.48	-
Mean NMB per patient ($)	35,287.98	31,465.14	34,218.98	33,163.44	1,069.00	45,715.08
	(24,870.97)	(22,176.63)	(24,117.54)	(23,373.59)	(753.43)	(32,219.98)
NMB ($)	114.3 billion	-	110.7 billion	-	3.6 billion	-
	(80.5 billion)		(78 billion)		(2.5 billion)	

*Note*: Mean visit costs include the average costs of visiting the ED and primary providers, and mean total costs per patient are the average overall costs patients incur in the simulation (this includes treatment costs and provider’s visit, ED visit, specialist visit, and productivity losses).

Abbreviations: SOC is the standard of care, LASOO+SOC depicts the inclusion of Lasoo health at the primary care level of patients with headaches’ care pathways, ED means emergency department, QALYs mean quality-adjusted life-years, NMB means net monetary benefit, and iNMB means incremental net monetary benefit. All costs are presented in Canadian dollars (CAD) with the equivalent in United States dollars (USD) provided in brackets.

**Figure 2.**
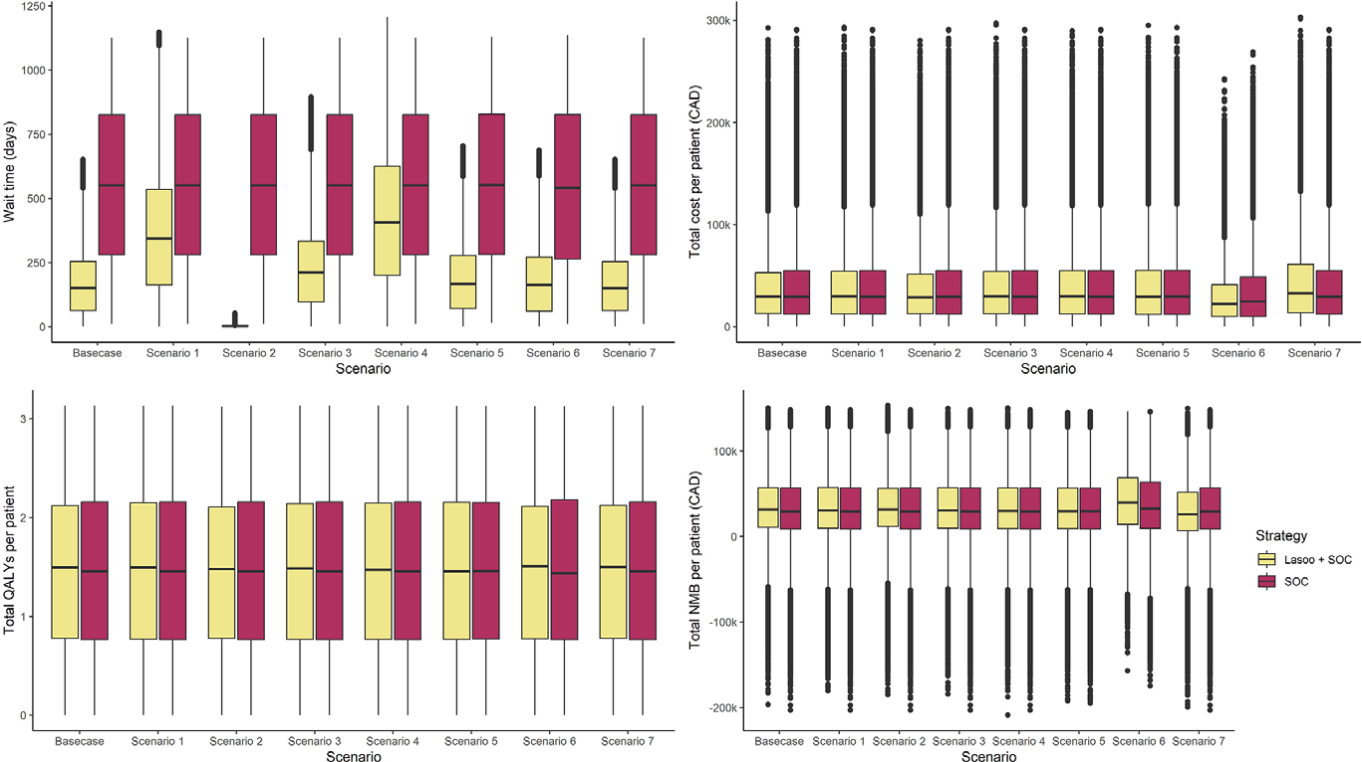
Boxplots of (A) wait times, (B) total costs, (C) quality-adjusted life-years (QALYs), and (D) net monetary benefit (NMB) per patient as it varies across different scenarios in the sensitivity analyses. Abbreviations: SOC is the standard of care, LASOO+SOC depicts the inclusion of Lasoo health at the primary care level of patients with headaches’ care pathways, ED means emergency department, QALYs mean quality-adjusted life-years, NMB means net monetary benefit, and iNMB means incremental net monetary benefit. The boxes represent the interquartile range of the respective results. *Note*: Scenario description: Scenario 1: The percentage of referred patients that consent to Lasoo Health was set to fifty percent; Scenario 2: The percentage of referred patients that consent to Lasoo Health was set to ninety-five percent; Scenario 3: The percentage of Lasoo Health reviews that require more information was set to fifty percent; Scenario 4: The percentage of Lasoo Health reviews that require an in-person specialist visit was set to fifty percent; Scenario 5: The proportion of a specialist’s workday that is available to patients with headaches’ appointments was set to twenty-five percent while ensuring the mean wait time for SOC matched the observed wait times in Alberta; Scenario 6: The referral rate for primary headaches was set to thirty percent; and Scenario 7: The effectiveness of headache treatments was set to twenty-five percent. Mean visit costs include the average costs of visiting the ED and primary providers, and mean total costs per patient are the average overall costs patients incur in the simulation (this includes treatment costs and provider’s visit, ED visit, specialist visit, and productivity losses).

Alternative assumptions were explored through scenario analyses (Table S4 and Figure 2). In all the scenarios analyzed, Lasoo + SOC was estimated to lead to shorter wait times, more QALYs, and less total costs than SOC. However, the degree to which this occurred varied depending on the parameters and model assumptions of each scenario. Increasing the percentage of referred patients with headaches who consent to Lasoo Health to 95 percent further reduced the wait time from a base case of 161.6 days to 4.69 days and increased the associated iNMB per patient from CAD 1,069 to CAD 1,337 (USD 753 to USD 942). Similarly, increasing the referral rate of primary headaches to 30 percent further increased the iNMB per patient to CAD 5,139 (USD 3,622). On the contrary, reducing the percentage of referred patients who consent to Lasoo Health to 50 percent is estimated to be associated with a mean wait time of 353 days and an iNMB per patient of CAD 609 (USD 428). Setting the percentage of specialist workday hours available for headache assessments to 25 percent is estimated to yield similar findings as in the base case; compared to the SOC, Lasoo + SOC reduces the wait times by 69 percent. The base case wait time associated with Lasoo + SOC is increased from 163 days to 227 days and 430 days when the percentage of Lasoo Health reviews requiring more information and the percentage of Lasoo Health reviews that require in-person visits are increased to 50 percent, respectively. Setting the effectiveness of headache treatments to 25 percent did not change the difference in wait times between Lasoo +SOC and SOC when compared to the base case. However, the difference in total costs was smaller when compared to the base case. Finally, threshold analysis of the impact of Lasoo Health consent rate showed that even with a consent rate as low as 5 percent, Lasoo +SOC is associated with a positive iNMB when compared to the SOC (Figure S1).

In total, compared to SOC, for all patients with headaches in Alberta over the span of five years, the base case early-stage analysis estimates that Lasoo + SOC could lead to societal cost savings of about CAD 3.1 billion (USD 2.2 billion) and an additional 7,900 QALYs, or an iNMB of CAD 3.6 billion (USD 2.5 billion), representing significant potential value of Lasoo and meriting further evaluation and technology development.

## Discussion

This analysis presents an eHTA of including Lasoo Health technology in the SOC for patients with headaches in Alberta, Canada. The base case simulation model was verified as an accurate representation of the current care pathways for patients with headaches in Canada, and the projected outcomes over a 5-year horizon were presented.

The early-stage analysis finds a significant difference in estimated wait times for specialist assessments of patients with headaches between SOC and Lasoo + SOC, with Lasoo + SOC estimated to yield considerably shorter wait times. Lasoo + SOC may also be associated with a decrease in total per-patient costs and higher health-related QALYs compared to SOC. Most of the cost savings come from a reduction in patient-level productivity losses with Lasoo + SOC, whereas savings in primary care provider’s or ED visits were limited. This highlights that most of the cost-saving value of Lasoo Health is expressed in societal value (shorter time to effective treatment) than in health system value (reduced healthcare utilization). Regardless of which strategy is employed, patients that are NEM will continue to have primary care provider’s and/or ED visits; thus, there are minimal differences in these outcomes.

Despite quite substantial differences in wait times between the two strategies, there were minimal differences in the accrued per-patient QALYs. This is because headaches are mostly nonfatal and have relatively constrained impacts on overall patient QALYs in the short term. However, headaches can be the cause of significant productivity losses through absenteeism and impaired effectiveness at work (37). Our results indicate that over 90 percent of the mean per-patient total cost is attributable to productivity losses. This is consistent with previous findings (38), where associated lost productivity costs were estimated to be about 93 percent of the total costs of migraine in Europe.

This early-stage analysis is based on secondary data sources in combination with expert consultations, which required several modeling assumptions. As such, there are some important structural and parameter limitations that we attempted to counter in part through applying more conservative (i.e., favoring the SOC) modeling assumptions, where possible. First, over-the-counter medication costs were not included, which patients with headaches may incur before diagnosis and getting an effective treatment plan. Although this cost would be incurred under both strategies, it is anticipated that more of it would be incurred under SOC because more patients with headaches get on effective treatment plans and at a faster rate under Lasoo + SOC. Second, it is likely that patients who had received Lasoo review assessments by specialists would be given some priority for in-person specialist visits, given that specialists can triage them through Lasoo Health intake. The model, however, assumes a first-come, first-served approach for patients in both the SOC and Lasoo + SOC strategies, without granting priority to those who consent to Lasoo Health. Third, patient no-shows or lateness to appointments, specialists’ nonavailability for prescheduled appointments, and nonadherence to treatment plans were not included in the simulation. Including these would provide a more comprehensive understanding of model assumptions and their impact on wait times and resource utilization as this could lead to loss of anticipated revenue (39;40). Fourth, for this analysis, the utilities on the number of MHD did not take into consideration the type of headache experienced. Two patients experiencing different types of headaches but with the same MHD may not necessarily have the same score. Also, reduction in MHD and thus improvement in utility may be heterogeneous across different headache types. As such, this assumption may not reflect sufficiently the impact that Lasoo could have on QALYs. However, our current analysis lacks precise data to reflect this heterogeneity adequately. Fifth, certain model inputs such as the number of neurologists and duration of specialist appointments in Alberta were based on expert opinion, which may not accurately reflect the current situation. However, should more accurate data become available, updating these parameters would have minimal effect on model results as the model was calibrated to replicate the current wait times in Alberta. Sixth, the 5-year simulation horizon adopted here may not be sufficient to capture the full impact of an eConsult software like Lasoo Health on chronic health conditions. This is because there may be an increased effect on health outcomes and costs beyond this timeframe. Therefore, long-term follow-up is required to evaluate the health and economic benefits of Lasoo Health over time with greater accuracy. However, certain model parameters such as the number of specialists were fixed throughout the simulation, and as such, longer time frames would have compromised the accuracy of this assumption. Seventh, for existing eConsult programs in Canada (41;42), the turnaround time for specialists’ assessments through these platforms is between a few days and a week regardless of the wait times for specialist assessment of the condition of interest, which may suggest that specialists spend time outside usual work hours on eConsults. However, to compare Lasoo + SOC to SOC, it was assumed that they both have identical work hours to isolate the impact of Lasoo Health without the contribution of other factors that could be beneficial for model outcomes. Last, the implementation or recurring costs of Lasoo Health were not included in the analysis due to a lack of data. As such, the results of this analysis should be interpreted with caution and data on implementation costs will be needed to further substantiate the impact of Lasoo Health. Despite these limitations, this analysis provides initial insight into the economic impact of incorporating Lasoo Health in headache care pathways for patients.

Based on the limitations of this analysis and the current state of health technology assessments of eConsult systems in the literature, we recommend that evaluative research on newly developed and already existing eConsult programs should be explored. Specifically, in headache management, data on factors such as the impact of eConsults on healthcare utilization, patient/doctor perspectives on acceptance, the integration of the platforms into existing medical health records, and health system costs should be collected to help with model validation (43;44). These factors are not clearly articulated in the literature but are likely to impact the adoption and efficiency of these programs (45).

## Conclusions

As part of an eHTA of Lasoo Health technology, we developed a DES model for headache management in Alberta to assess the potential cost-effectiveness of incorporating Lasoo Health in the SOC pathways for patients with headaches. Our results indicate that Lasoo Health could potentially reduce wait times, improve health outcomes for patients with headaches, and save costs. The cost savings were mostly attributed to a reduction in productivity losses. This analysis finds there is potential value for the incorporation of eConsult programs like Lasoo Health into the care pathways for chronic conditions like headaches, as they may reduce patient-level burden primarily through decreased wait times.

The economic model presented here provides a comprehensive framework to explore the potential economic value of eConsult programs like Lasoo Health in the management of health conditions with long wait times for specialist care. Although the focus of this analysis is on the impact of Lasoo Health on headaches, the results could be generalized to other chronic conditions with similar care pathways. Developed at an early stage of the software’s life cycle, our model offers the benefit of guiding data collection in future studies (46, 47), particularly highlighting certain parameters that should be collected during Lasoo Health’s implementation.

## Supporting information

10.1017/S0266462326103547.sm001 Koles et al. supplementary material Koles et al. supplementary material

## Supplementary material

The supplementary material for this article can be found at http://doi.org/10.1017/S0266462326103547.
