# Supplementary material for: Early health technology assessment of a primary care tool for the diagnosis and management of headaches in Alberta, Canada
Source: Int J Technol Assess Health Care. 2026 Feb 20;42(1):e35. doi: 10.1017/S0266462326103547 (PMC13071843; doi:10.1017/S0266462326103547)
Supplement: Koles et al. supplementary material [file S0266462326103547sup001.docx]

**Supplementary document**

**Table S1: Basecase model parameters**

| Parameter | Value | Source |
| --- | --- | --- |
| Percentage of headache patients that experience migraine (%) | 54 | (1) |
| Percentage of headache patients that experience tension type headache (%) | 10 | (1) |
| Percentage of headache patients that experience secondary headaches (%) | 35 | (1) |
| Percentage of headache patients that experience cluster headaches (%) | 1 | (1) |
| Mean age of migraine headache patients (sd) | 42.17 (13.14) | (2) |
| Mean age of tension type headache patients (sd) | 44.78 (12.78) | (2) |
| Mean age of cluster headache patients (sd) | 36.13 (9.6) | (2) |
| Mean age of secondary headache patients (sd) | 51.39 (15.5) | (2) |
| Monthly headache days (migraine) | 7-19 | (3) |
| Monthly headache days (tension headache) | 3-28 | (4) |
| Monthly headache days (cluster headache) | 14-28 | (5) |
| Monthly headache days (secondary headache) | 3 - 20 | (4) |
| Capacity and service times | | |
| Number of specialists | 100 | Expert opinion |
| Percentage of specialists that do general neurology and headache assessment (%) | 50 | Expert opinion |
| Number of primary care physicians | 5540 | (6) |
| Duration of a primary care doctor’s visit (minutes) | 25 - 30 | Expert opinion |
| Duration of a specialist visit (minutes) | 30 – 40 | Expert opinion |
| Duration of specialist review of Lasoo Health intake and diagnosis (minutes) | 10 - 15 | Expert opinion |
| Duration of an emergency department visit (minutes) | 60 - 75 | Expert opinion |
| Number of patients seen in a week by a primary care physician | 100 | (7) |
| Percentage of primary care doctors’ visits that are headache patients (%) | 1.5 - 4 | (8) |
| Annual number of missed workdays due to headaches (sd) | 46 (84.6) | (3) |
| Time between when referral is sent, received and when available appointment is communicated (days) | 14 - 28 | (9) |
| Mean time to secure a primary care doctor’s appointment (days) | 3 | (10) |
| Mean time in days it takes a patient to complete web-based Lasoo Health intake form | 1-2 | Assumed |
| Treatment effectiveness | | |
| Effectiveness of migraine treatments | 0.5 | (11) |
| Effectiveness of tension type headache treatments | 0.5 | (11, 12) |
| Effectiveness of cluster headache treatments | 0.46 | (13) |
| Effectiveness of secondary headache treatments | 0.5 | (14) |
| Costs ($) | | |
| Cost of primary care doctor’s visit | 35 | (15) |
| Cost of specialist visit | 75 | (15) |
| Cost of specialist review of Lasoo Health intake | 20 | (15) |
| Cost of emergency department visit | 101.73 | (15) |
| Annual cost of headache treatments | 167 – 12,768 | (16) |
| Cost of a missed day at work | 232 | (17, 18) |
| Utilities | | |
| Monthly headache days (0 -3) | 0.7573 | (19) |
| Monthly headache days (4-9) | 0.6449 | (19) |
| Monthly headache days (10 - 14) | 0.6764 | (19) |
| Monthly headache days (15 -19) | 0.6420 | (19) |
| Monthly headache days (20 – 23) | 0.5916 | (19) |
| Monthly headache days (24-28) | 0.5040 | (19) |
| Probabilities | | |
| Probability of referrals for primary headaches | 0.02 | (20) |
| Probability of referrals for secondary headaches | 1 | (21) |
| Probability of receiving effective treatment during primary care doctors visit | 0.1 | Assumed |
| Probability of consent to Lasoo Health | 0.75 (0.5 – 0.95) | (22, 23) |
| Probability that specialist would not need further information after review of Lasoo Health intake | 0.95 | (24) |
| Probability that specialist will require further information after review of Lasoo Health intake | 0.025 | (24) |
| Probability that specialist would require in person or virtual visit after review of Lasoo Health intake | 0.025 | (24) |

**Table S1:** Model outcomes for Lasoo Health, SOC and the difference between them for the base case analysis from a healthcare perspective. Abbreviations: SOC is the standard of care, LASOO+SOC depicts the inclusion of Lasoo health at the primary care level of patients with headaches care pathways, ED means emergency department, QALYs mean quality adjusted life years, NMB means net monetary benefit, and iNMB means incremental net monetary benefit. All costs are presented in Canadian dollars (CAD) with the equivalent in United States dollars (USD) provided in brackets.

| Outcome | Lasoo + SOC | | SOC | | Difference | |
| --- | --- | --- | --- | --- | --- | --- |
|  | Mean | SD | Mean | SD | Mean | SD |
| Average wait time  (days) | 162.68 | 110.13 | 555.54 | 313.81 | -392.87 | 332.57 |
| Mean headache days per month  (days) | 8.50 | 4.38 | 9.46 | 4.76 | -0.96 | 6.47 |
| Percentage of cohort effectively managed  (%) | 70.91 | - | 53.23 | - | 17.68 |  |
| Mean number of primary care provider visits per  patient | 7.44 | 5.69 | 7.48 | 5.49 | -0.04 | 7.91 |
| Mean number of ED visits  per patient | 6.19 | 5.51 | 6.41 | 5.34 | -0.22 | 7.67 |
| Mean total missed workdays  per patient | 134.94 | 134.03 | 149.18 | 146.64 | -14.24 | 198.67 |
| Mean treatment costs per patient ($) | 8,047.85  (5,672.12) | 10,259.50  (7,230.89) | 6,123.44  (4,315.80) | 9,756.10  (6,876.09) | 1,924.41  (1,356.32) | 14,157.64  (9,978.30) |
| Mean Productivity losses per patient ($) | 0 | - | 0 |  | - | 0 |
| Mean total costs per patient ($) | 8,924.6  (6,290) | 10,259.5  (7,287) | 6,123.4  (4,952.5) | 9,756.1  (6,855.8) | 1,924.4  (1,337.6) | 14,196.2  (10,005.5  ) |
| Total costs ($) | 28.9 billion  (20,377,012,901) | - | 22.7 billion  (16,024,928,857) | - | 6.2 billion  (4,352,084,044) | - |
| Mean total QALYs per  patient | 1.44 | 0.78 | 1.44 | 0.80 | 0.00 | 1.12 |
| Total accrued QALYs | 4.7billion | - | 4.7billion | - | 7,976.48 | - |
| Mean NMB per patient ($) | 63,212.8  (24,870.97) | 33,465.9  (22,176.63) | 65,072.7  (24,117.54) | 34,985.7  (23,373.59) | -1,859.9  (753.43) | 48,414.6  (32,219.98) |
| NMB ($) | 204.8billion  (144.3 billion) | - | 210.6 billion  (148.4 billion) | - | -5.8 billion  (-4.1 billion) | - |

Definitions: Mean visit costs include the average costs of visiting the ED and primary providers, Mean total costs per patient is the average overall cost patients incur in the simulation (this includes treatment costs and providers visit, ED visit, specialist visit, and productivity losses).

Table S3: Results of multiple realization of DES model.

| **Outcome** | **Lasoo + SOC** | | **SOC** | | **Difference** | |
| --- | --- | --- | --- | --- | --- | --- |
|  | **Mean** | **SD** | **Mean** | **SD** | **Mean** | **SD** |
| Average wait time (days) | 166.90 | 0.74 | 552.01 | 0.54 | -385.11 | 0.81 |
| Mean headache days per month | 8.50 | 0.00 | 9.46 | 0.00 | -0.96 | 0.00 |
| Proportion of cohort effectively managed | 71.01 | 0.03 | 53.22 | 0.03 | 17.79 | 0.04 |
| Number of Physician visits per patient | 7.44 | 0.00 | 7.48 | 0.00 | -0.04 | 0.01 |
| Number of ED visits per patient | 6.19 | 0.00 | 6.41 | 0.00 | -0.22 | 0.01 |
| Visit costs per patient ($) | 876.86 | 0.48 | 903.26 | 0.42 | -26.40 | 0.66 |
| Treatment costs per patient ($) | 8,063.84 | 6.19 | 6,125.51 | 5.35 | 1,938.33 | 6.41 |
| Productivity losses per patient ($) | 25,527.53 | 13.72 | 28,483.10 | 15.68 | -2,955.57 | 24.29 |
| Total costs per patient ($) | 34,468.23 | 15.47 | 35,511.86 | 14.25 | -1,043.64 | 25.98 |
| QALYs per patient | 1.58 | 0.00 | 1.57 | 0.00 | 0.00 | 0.00 |

Table S4: Scenario analyses varying different model parameters to assess the impact on analyses results.

| Scenario | Mean wait time (days) | | | Percentage effectively managed (%) | | | Total cost per patient ($) | | | QALYs per patient | | | iNMB  ($) |
| --- | --- | --- | --- | --- | --- | --- | --- | --- | --- | --- | --- | --- | --- |
|  | Lasoo +SOC | SOC | Difference | Lasoo + SOC | SOC | Difference | Lasoo + SOC | SOC | Difference | Lasoo + SOC | SOC | Difference |  |
| Consent to Lasoo is 50% | 352.69 | 555.54 | -202.88 | 61.98 | 53.23 | 8.76 | 37,463.15 | 37,880.55 | -417.39 | 1.44 | 1.44 | 0.00 | 608.64 |
| Consent to Lasoo is 95% | 4.69 | 555.54 | -550.85 | 79.98 | 53.23 | 26.75 | 36,211.47 | 37,880.55 | -1,669.08 | 1.44 | 1.44 | 0.00 | 1,337.84 |
| The probability that a specialist will require further information after review of Lasoo is 50% | 226.82 | 555.54 | -328.72 | 63.84 | 53.26 | 10.62 | 37,442.47 | 37,880.55 | -438.083 | 1.44 | 1.44 | 0.00 | 605.94 |
| The probability that a specialist will require in person or virtual visit after review of Lasoo is 50% | 430.10 | 555.54 | -125.44 | 57.24 | 53.26 | 4.02 | 37,442.47 | 37,880.55 | -438.08 | 1.44 | 1.44 | 0.00 | 153.46 |
| 25% of specialists workday available to headache patients | 179.16 | 556.76 | -377.59 | 70.13 | 53.19 | 16.94 | 36,900.56 | 37,868.93 | -968.36 | 1.44 | 1.44 | 0.00 | 1,053.46 |
| Referral rate of primary headaches is set to 30% | 171.12 | 548.95 | -377.84 | 77.46 | 44.34 | 33.11 | 28,602.66 | 33,721.79 | -5,119.13 | 1.44 | 1.44 | 0.00 | 5,139.23 |
| Effectiveness of headache treatments set to 25% | 162.67 | 555.54 | -392.87 | 70.90 | 53.22 | 17.67 | 45,227.69 | 45,124.28 | 103.41 | 1.44 | 1.44 | 0.00 | 73.99 |

Figure S1: Threshold analysis of the impact of consent rate on the cost effectiveness of Lasoo Health when compared to the standard of care.


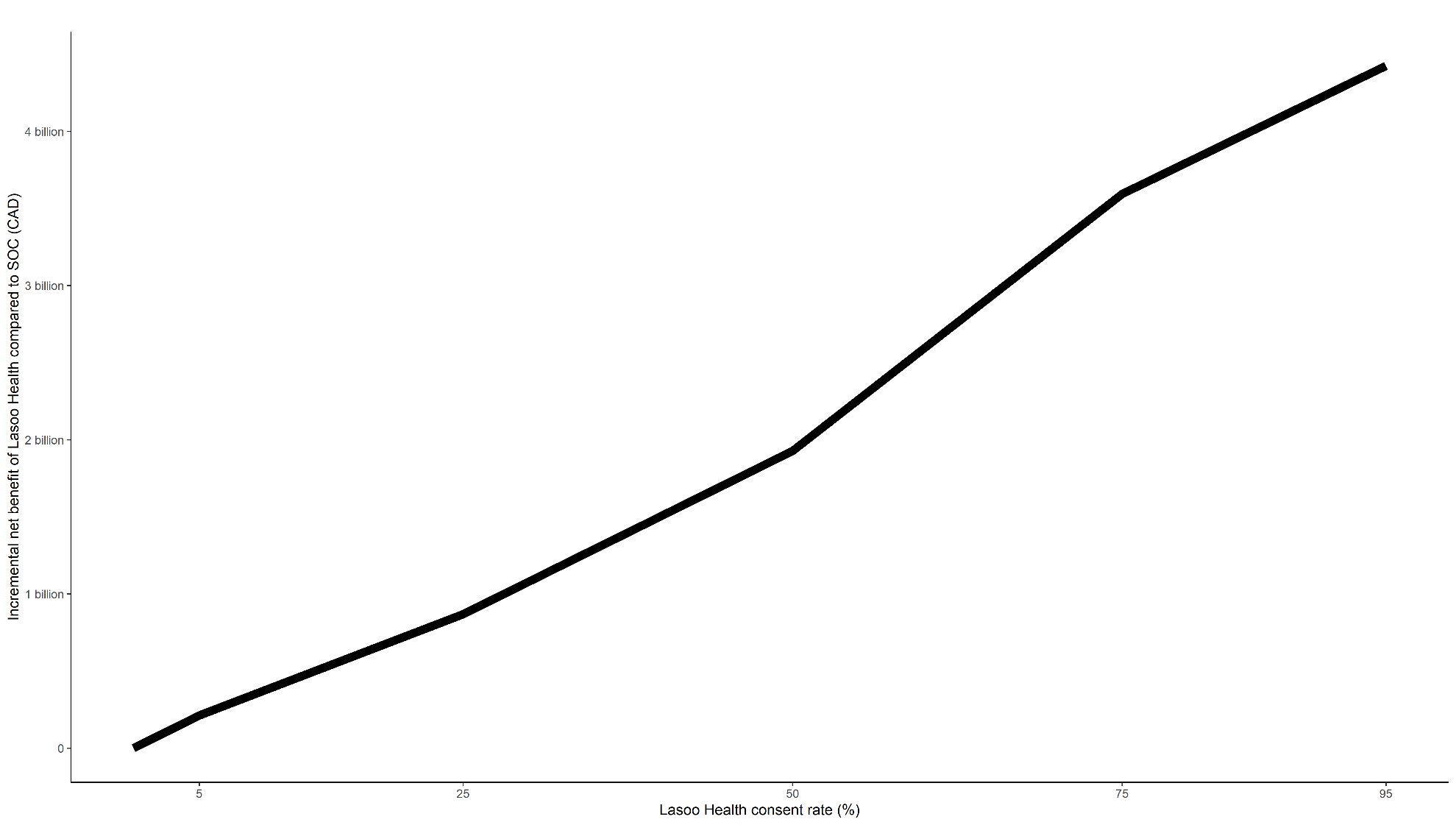


## References

1. Friedman BW, Hochberg ML, Esses D, Grosberg B, Corbo J, Toosi B, et al. Applying the International Classification of Headache Disorders to the emergency department: an assessment of reproducibility and the frequency with which a unique diagnosis can be assigned to every acute headache presentation. Annals of emergency medicine. 2007;49(4):409-19. e9.

2. Wang Y, Zhou J, Fan X, Li X, Ran L, Tan G, et al. Classification and clinical features of headache patients: an outpatient clinic study from China. J HEADACHE PAIN. 2011;12:561-7.

3. Amoozegar F, Khan Z, Oviedo-Ovando M, Sauriol S, Rochdi D. The burden of illness of migraine in Canada: new insights on humanistic and economic cost. Can J Neurol Sci. 2022;49(2):249-62.

4. Specialistlink. Primary care pathway: headache and migraine 2024 [Available from: <https://www.specialistlink.ca/assets/pdf/CZ_Neurology_HeadacheMigraine_Pathway.pdf>.

5. Diener HC, May A. Drug treatment of cluster headache. Drugs. 2022;82(1):33-42.

6. McDonald T, Jessie Hart S, Lee A G. The Supply of Primary Care Physicians in Alberta 2018-2022 – A Dire Description. Canadian Health Policy. 2023.

7. Statista. Median number of patients seen during a typical workweek by primary care physicians in Canada in 2019, by jurisdiction Statista website2020 [cited 2024. Available from: Median number of patients seen during a typical workweek by primary care physicians in Canada in 2019, by jurisdiction.

8. Becker L, Iverson DC, Read F, Calogne N, Miller R, Freeman W. A study of headache in North American primary care: Report for the Ambulatory Sentinel Practice Network. 1987.

9. AlbertaReferralDirectory. Alberta Health services - Calgary zone 2024 [Available from: <https://albertareferraldirectory.ca/PublicSearchController?direct=displayViewServiceAtFacility&serviceAtFacilityId=1131917&pageNumberToDisplay=1&sortOrder=9&publicSearch=true&backToPage=solrSearchScreen1>.

10. Healthing.ca. Half of Canadians can't get a doctors' appointment 2024 [Available from: <https://www.healthing.ca/wellness/family-doctor-shortage-poll-angus-reid>.

11. Lampl C, MaassenVanDenBrink A, Deligianni CI, Gil-Gouveia R, Jassal T, Sanchez-del-Rio M, et al. The comparative effectiveness of migraine preventive drugs: a systematic review and network meta-analysis. The journal of headache and pain. 2023;24(1):56.

12. Murphy C HS. Chronic Headaches Internet: StatPearls Publishing;; 2024 [Available from: <https://www.ncbi.nlm.nih.gov/books/NBK559083/>.

13. Fogh‐Andersen IS, Sørensen JCH, Jensen RH, Knudsen AL, Meier K. Treatment of chronic cluster headache with burst and tonic occipital nerve stimulation: A case series. Headache: The Journal of Head and Face Pain. 2023;63(8):1145-53.

14. Zeeberg P, Olesen J, Jensen R. Discontinuation of medication overuse in headache patients: recovery of therapeutic responsiveness. Cephalalgia. 2006;26(10):1192-8.

15. AlbertaMedicalAssociation. Fee Navigator 2024 [Available from: <https://www.albertadoctors.org/fee-navigator/hsc/03.03A>.

16. CADTH. CADTH Reimbursement Reviews and Recommendations. Canadian Journal of Health Technologies. 2019.

17. StatisticsCanada. Income of individuals by age group, sex and income source, Canada, provinces and selected census metropolitan areas 2024 [cited 2024. Available from: <https://www150.statcan.gc.ca/t1/tbl1/en/tv.action?pid=1110023901>.

18. Ilersich L. An economic analysis of Sumatriptan for acute migraine. 1997.

19. Mistry H, Naghdi S, Underwood M, Duncan C, Madan J, Matharu M. Competing treatments for migraine: a headache for decision-makers. J HEADACHE PAIN. 2023;24(1):162.

20. Latinovic R, Gulliford M, Ridsdale L. Headache and migraine in primary care: consultation, prescription, and referral rates in a large population. Journal of Neurology, Neurosurgery & Psychiatry. 2006;77(3):385-7.

21. Becker WJ, Findlay T, Moga C, Scott NA, Harstall C, Taenzer P. Guideline for primary care management of headache in adults. Canadian Family Physician. 2015;61(8):670-9.

22. Ackerman SL, Gleason N, Shipman SA. Comparing patients’ experiences with electronic and traditional consultation: results from a multisite survey. Journal of General Internal Medicine. 2020;35:1135-42.

23. Joschko J, Liddy C, Moroz I, Reiche M, Crowe L, Afkham A, et al. Just a click away: exploring patients’ perspectives on receiving care through the Champlain BASETM eConsult service. Fam Pract. 2018;35(1):93-8.

24. Thompson MA, Fuhlbrigge AL, Pearson DW, Saxon DR, Oberst-Walsh LA, Thomas JF. Building eConsult (electronic consults) capability at an academic medical center to improve efficiencies in delivering specialty care. Journal of Primary Care & Community Health. 2021;12:21501327211005303.
